# High HLA Sensitization After Early Renal Allograft Vascular Thrombosis

**DOI:** 10.3389/ti.2025.14457

**Published:** 2025-06-27

**Authors:** María José Pérez-Sáez, Jordi Comas, Edoardo Melilli, Francesc Moreso, Lluis Guirado, Anna Vila, Fritz Diekmann, Eduard Palou, Jaume Tort, Dolores Redondo-Pachón, Marta Crespo

**Affiliations:** ^1^ Nephrology Department, Hospital del Mar, RICORS2040 RD21/0005/0022, Barcelona, Spain; ^2^ Hospital del Mar Research Institute, Barcelona, Spain; ^3^ Organització Catalana de Trasplantaments, Barcelona, Spain; ^4^ Nephrology Department, Hospital Universitari de Bellvitge, L’Hospitalet de Llobregat, Barcelona, Spain; ^5^ Nephrology Department, Hospital Universitario General Vall d’ Hebron, Barcelona, Spain; ^6^ Nephrology Department, Fundació Puigvert, Institut de Recerca Sant Pau (IR Sant Pau), RICORS2040, Barcelona, Spain; ^7^ Nephrology Department, Hospital Universitari Germans Trias i Pujol, RICORS2040, Barcelona, Spain; ^8^ Nephrology Department, Hospital Clínic, Barcelona, Spain; ^9^ Laboratory of Histocompatibility of Catalunya, Barcelona, Spain

**Keywords:** immunosuppression, sensitization, HLA, PRA, vascular thrombosis

Dear Editors,

Renal allograft thrombosis represents the most frequent cause of early allograft loss in our media, only preceded by the death of the recipient with a functioning graft in recent years [1, 2]. For a long time, the immediate surgical loss of the graft has been accompanied by graft removal and immediate immunosuppression cessation. However, there is scarce information about the impact these early failed kidney transplants (KT) might have on mid- and long-term recipient human leukocyte antigen (HLA) sensitization, with small studies revealing a potential HLA sensitization phenomenon if patients wait a long time before the new transplant [3, 4]. Besides the graft itself and the time for HLA antigens allorecognition, it seems biologically plausible that the remaining donor vascular tissue after graft nephrectomy could serve as a font for recipient HLA sensitization. Although recent guidelines encourage clinicians to maintain immunosuppression if the patient is a candidate for another transplant, even when the graft duration inside the recipient may be only a few hours [5], other and also recent consensus documents still recommend withdrawing immunosuppression after renal allograft surgical failure [6]. Outcomes in recipients who experience early graft loss are usually worse, with only 50% being re-listed [7], and with lower graft survival probability in their following transplantation if they got highly sensitized [8]. In addition, there are no clear strategies or policies to prioritize these recipients once they are relisted for transplantation. In Catalonia, recipients maintain their original time on dialysis (not considering the failed transplantation as a new starting point), but without any other exceptional measures to speed up the new transplant.

Using data from the Renal Catalan Registry, we aimed to analyze the calculated panel reactive antibody (cPRA) class I and class II evolution after an early allograft vascular thrombosis in a cohort of KT recipients. For this purpose, we performed a retrospective analysis including data from recipients of their first KT performed in Catalonia between 2015 and 2023 who suffered from an allograft loss because of vascular thrombosis within the first 30 days after transplantation. To analyze cPRA evolution, we selected those patients re-included on the KT waiting list for a second transplantation after the thrombosis (and, therefore, with available cPRA data). We excluded multiorgan transplants and recipients with a pretransplant cPRAI+II >90%. Trying to isolate the thrombosis as the sole event of sensitization, we also excluded patients who received blood transfusions after the transplant, merging our database with the Catalan Blood and Tissue Bank transfusions data. We analyzed the trend in cPRA after graft loss during the first year after relisting in those who did not get a second transplant. The MFI threshold for defining unacceptable antigens was set as follows: 1) above 750, and 2) exceeding the bead-specific threshold for the lowest bead within the same locus. The loci included in the cPRA calculation were HLA-A, B, C, DRB1, and DQB1. DRB3/4/5, DQA1, DPB1 and DPA1 loci were not included in the calculation.

During the study period, 5484 1st KT were performed in Catalonia. Of those, 249 failed within 1 month after transplantation (40 patients died and 209 lost their grafts). One hundred twenty-two patients experienced a vascular allograft thrombosis, representing 58.4% of early graft losses (<30 days), and 2.2% of the overall graft losses. Seventy-six cases of these thrombosis (62%) happened within the first 72h post-transplant–immediate thrombosis-; and 46 from day 4 to day 30 after transplantation–early thrombosis-. Recipients were 64.8% male and the mean age was 61.7 years old. Renal grafts came from donors after brain death in 53.3% of the cases; 33.6% from controlled cardiocirculatory death donors; 9% from living donors; and 4.1% from uncontrolled cardiocirculatory death donors. After the transplant, 52.5% of the recipients received blood transfusions. We analyzed cPRA data from 45 patients (32 with immediate thrombosis and 13 with early thrombosis) who had pre-KT cPRA 0% [interquartile range 0–23], who did not receive any blood transfusion after transplant, and who were re-included on the KT waiting list until December 2023. All subjects discontinued the antimetabolite immunosuppressive drug immediately following transplantectomy. Regarding tacrolimus, 33 subjects discontinued the drug early after transplantectomy (mean time: 1.6 days), while 12 patients continued tacrolimus for a longer period (at least 1 year). Steroids were discontinued shortly after graft loss in 34 patients (mean time: 2.5 days), whereas 11 subjects maintained a minimum dose of prednisone for a longer duration, alongside tacrolimus.

One month after transplant (median 28 days; interquartile range [17–39], n = 19), median cPRA increased from 0% to 28%, interquartile range [0–79]; after 3 months (88.5 days [72–100], n = 14) to 75.5% [52–95]; and after 6 months (196 days [174–198], n = 9) cPRA reached 99% [93–100] (p = 0.037), Figure 1. From the total 45 cases included in the study, last-follow-up cPRA was 87% [0–100]. In addition, 10.8% of these recipients needed to be included in a national prioritization program to find an HLA compatible donor for their following transplant because of a cPRA ≥98%.

**FIGURE 1:**
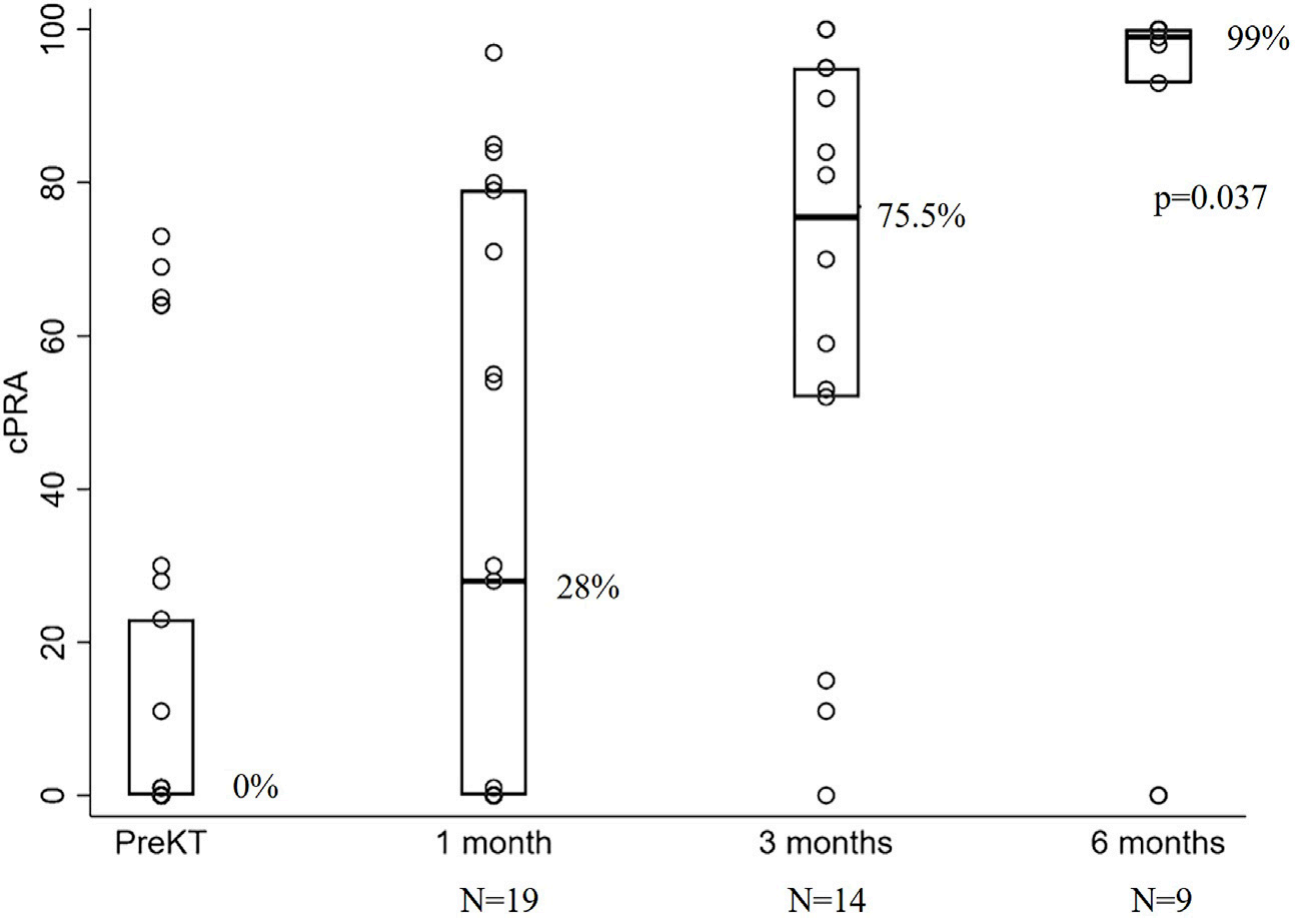
Strip plot showing cPRA evolution after vascular kidney allograft thrombosis. N represents the number of patients with available cPRA data at each time point.

Regarding relisting and retransplantation rate, as of September 2024, 75.4% of the 122 cases of thrombosis-related graft failure were relisted (81/122 (66.4%) cases during the first year after graft failure), and 60.7% underwent retransplantation (35/122 (28.7%) during the first year after relisting. The median time between listing and transplantation was 397 days (interquartile range [IQR]: 120–670). For all second deceased-donor kidney transplants performed in Catalonia between 2015 and 2023, the median wait time was 404.5 days (IQR: 116–1015).

After this analysis, the Catalan renal allocation program changed their policy and prioritized these patients for a second transplant. Currently, recipients with a history of graft thrombosis are prioritized for their second transplant using the first available deceased-donor kidney in Catalonia, provided it is not allocated to other prioritization programs such as simultaneous pancreas-kidney, pediatric, or highly sensitized recipients. With this policy, patients should be transplanted before they become highly sensitized.

In conclusion, despite the low number of patients, this study shows that solely renal allograft vascular thrombosis represents a strong HLA sensitization event for the recipient and potential candidate for a second transplant. These results should be considered for prioritization allocation policies and clinical practice to decide on immunosuppression reduction/cessation after graft loss.

## Data Availability

The raw data supporting the conclusions of this article will be made available by the authors, without undue reservation.
